# From Field Effect Transistors to Spin Qubits: Focus on Group IV Materials, Architectures and Fabrications

**DOI:** 10.3390/nano15221737

**Published:** 2025-11-17

**Authors:** Nikolay Petkov, Giorgos Fagas

**Affiliations:** 1 Physical Sciences Department, Munster Technological University, Bishopstown Campus, T12 P928 Cork, Ireland; 2 Tyndall National Institute, University College Cork, T12 R5CP Cork, Ireland; georgios.fagas@tyndall.ie

**Keywords:** quantum computing, one-dimensional structures, silicon, germanium

## Abstract

In this review, we focus on group IV one-dimensional devices for quantum technology. We outline the foundational principles of quantum computing before delving into materials, architectures and fabrication routes, separately, by comparing the bottom-up and top-down approaches. We demonstrate that due to easily tunable composition and crystal/interface quality and relatively less demanding fabrications, the study of grown nanowires such as core–shell Ge-Si and Ge hut wires has created a very fruitful field for studying unique and foundational quantum phenomena. We discuss in detail how these advancements have set the foundations and furthered realization of SETs and qubit devices with their specific operational characteristics. On the other hand, top-down processed devices, mainly as Si fin/nanowire field-effect transistor (FET) architectures, showed their potential for scaling up the number of qubits while providing ways for very large-scale integration (VLSI) and co-integration with conventional CMOS. In all cases we compare the fin/nanowire qubit architectures to other closely related approaches such as planar (2D) or III–V qubit platforms, aiming to highlight the cutting-edge benefits of using group IV one-dimensional morphologies for quantum computing. Another aim is to provide an informative pedagogical perspective on common fabrication challenges and links between common FET device processing and qubit device architectures.

## 1. Introduction

Quantum effects have been known for more than a century throughout the fast development of quantum mechanics. While many technologies such as the operation of lasers or magnetic resonance imaging exploit energy quantization, a main concept of quantum mechanics, there are other fundamental quantum principles that are awaiting to be turned into devices and systems with real practical use. For example, when two quantum particles are prepared in a superposition (entangled) state, they remain connected; therefore, observing the state of one immediately affects the other, even when separated by great distances. Although well described by Bell’s inequalities equations [1], this effect, called by Albert Einstein “spooky action at a distance”, was initially accepted as an oddity of the quantum mechanics and not a physical phenomenon that can lead to practical devices. Nonetheless, this is the main principle for realizing quantum computation with a basic element known as a quantum bit, or qubit, which in analogy to a classical bit contains information. The realization of qubits can use different quantum mechanical degrees of freedom such as charge, spin, photon polarization, magnetic flux, etc. Their physical representation can be in the form of single atoms in ion traps [2], nitrogen vacancies in diamond [3], superconducting circuits [4], spin-based qubits in semiconductor devices [5] and other platforms.

Based on these main platforms, in the past two decades, quantum computing has evolved from a speculative playground into an industrial race; many of the key players such as Intel, IBM, Google and others [6] are demonstrating functional quantum computing platforms. While the industrial race to quantum advantage has started, many countries are investing in quantum computing research with a clear tendency to increase the number of published works and have real impact on quantum science and engineering. The Scopus database shows more than 4000 published papers per year for the last few years. While different quantum computing platforms are being explored each with their specific advantages and challenges, it is still unclear which platform will win the quantum computing race [7]. Undoubtfully, the road to fully operational quantum computers will need to face a reality check against challenges such as fault-tolerant operation with a larger number of qubits, as well as scalable fabrication and co-integration with conventional complementary metal oxide semiconductor (CMOS) logic devices. A viable option to achieve scalable dense integration and to reduce future costs will be to have qubits operating at a higher temperature (4 Kelvin or above) and to adapt the existing paradigm of very large-scale integration (VLSI) in manufacturing that has been continuously perfected throughout the CMOS area [8].

The electron/hole or indeed the nuclear spin states provide a two-level system that is excellent for demonstrating the main concepts underpinning the qubits operation [5]. The realization of these systems has become possible in the last decade due to (i) the extensive knowledge accumulated in the areas of mesoscopic physics—from the theory of quantum confinement and the semiconductors’ band structure engineering to experimental demonstrations of main quantum phenomena such as Coulomb blockade in quantum dots (QDs) and of quantum device operation such as single electron transistors (SETs) or Josephsen junction transistors (JJTs); (ii) the advancements in the growth and fabrication of devices using multiples of gates on purposely engineered substrates that can act as hosts for gate-defined QDs and can harvest spin-based quantum properties and interactions; and finally (iii) the demonstration of spin exchange in semiconductor qubits on a large scale in devices fabricated by foundry-based processing routines, looking at reliability in the qubits operation including long-range interactions, and addressing challenges with fan-out of control, read-out, and possible all-electronic control [8].

On the other hand, the last two decades have seen extensive progress in one-dimensional (also known as nanowires, nanobelts or nanofins) semiconductors research. This covers both growth and device fabrication by bottom-up vs. top-down paradigms [9] and applications in gas [10] and biochemical sensing [11], energy storage [12], thermoelectric [13] and electromechanical operations [14]. Applications also explore the realm of neuron-nanowire interfaces and brain-inspired information processing [15]. We note that there is also a considerable progress made also with III–V (InAs and InSb) nanowires in demonstrating important quantum phenomena, a topic that has been recently reviewed [16].

Although there are extensive reviews describing semiconductor spin qubits and their role among other platforms [5,7], as well as more topical reviews about qubits based on one-dimensional structures [16] and group IV materials [8], there is no review that is aiming to comprehensively describe the link between the spin qubit devices, the challenges in their fabrications and the main fundamental physical properties found by relatively simple devices such as SETs. Moreover, an informative pedagogical perspective, mapped from conventional through fin/nanowire FETs with new channel materials such as Ge to the group IV quantum devices development has not been given before.

Herein, we start with the main concepts and material platforms used for group IV quantum devices, and then we follow with a discussion on the routes for device fabrication and the transition from the fin/nanowire FET devices to devices that demonstrate single electron/hole transport. We discuss in detail how these advancements have set the foundations and furthered realization of SETs and qubit devices with their specific operational characteristics. In all cases we compare with other closely related approaches such as planar (2D) or III–V qubit platforms, aiming to highlight the cutting-edge benefits of using one-dimensional (fin and nanowires) architectures with group IV materials for quantum computing.

## 2. Main Architectures and Material Platforms

**Figure 1:**
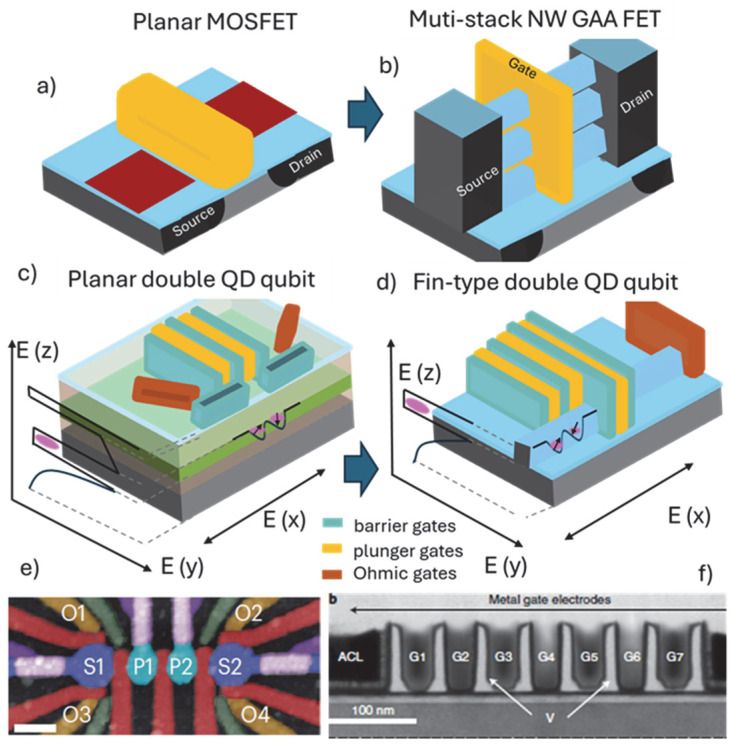
Schematics of device used in conventional FET technology and corresponding qubit architectures: (**a**) planar MOSFET and (**b**) vertically stacked nanowire (NW) FETs; devices used for gate-defined qubits: (**c**) planar quantum well (QW, green) and (**d**) nanowire/fin (blue) architectures for hosting QDs. QDs’ vertical confinement is illustrated in the plots of E (z) and lateral confinement is illustrated in the E (x/y)-plane provided by set of gates as described in the main text. Note the main transition from conventional FETs to qubit devices is in the higher complexity and number of gates needed for QDs definition and electron/hole spins localization. Historically, the planar (2D) architectures (**a**,**c**) evolved into non-planar (3D) architectures (**b**,**d**). Example electron microscopy images depicting multiple gates for the (**e**) planar QW [18] and (**f**) non-planar (3D) finFET qubit devices [19], respectively. In (**e**) S1 and S2 are sensor dots, P1 and P2 are plunger gates controlling the operation of the double QD and red and green are barrier gates. In (**f**) G1–G7 are alternating barrier and plunger gates over a Si fin.

For decades, the workhorse of conventional logic devices has been the planar metal-oxide semiconductor FET (MOSFET) architecture (Figure 1a). The planar (2D) device analogs (Figure 1c) are now perhaps the most common semiconductor spin qubit devices studied. The gate-defined QDs, hosted in strained Si, Ge and III–V layers, epitaxially grown as heterostructures, have been reviewed on several occasions, and the readers are directed to a comprehensive review by G. Burkard et al. [5] covering fundamental mesoscopic physics experiments and spin–spin interactions for qubits operation. Arguably, the most important technological advancements that enabled those demonstrations was the ability to grow high-crystal quality quantum well (QW) layers with enhanced mobility, forming 2D electron/hole gas (2DEG or 2DHG) systems [17]. This was complemented by the capacity of patterning high-density metal gates to define the QDs. With regard to group IV hosts, notable examples include growth of SiGe/Si-QW/SiGe or GeSi/Ge-QW/GeSi on Si carrier wafer substrates and the use of electron beam lithography (EBL) for patterning multiple layers of metal gates, electrically insulated and in any desired and sometimes very complex arrangements (Figure 1e,f) [18,19].

In analogy to the evolution of the planar Si MOSFETs devices to multi-gate architectures and the establishment of the finFETs technology [20], it might be expected that the fin/nanowire-based qubit platform will also have its role. A transition from planar QW-type quantum devices to non-planar architecture is a natural pathway. The logic devices roadmap has seen the development of multi-gate (π- or Ω-gate) finFETs, and currently the multi-stacked nanowire gate-all-around FETs (Figure 1b) have emerged as the most practical and cost-effective alternatives in achieving required performance for downsizing Si CMOS. The challenges faced and the knowledge accumulated during this process are linked to the important steppingstones for the recent demonstration of the first Si-fin spin qubits platform fabricated in a 300 mm semiconductor fabrication facility using all the manufacturing tools for the conventional CMOS devices (Figure 1f). However, we contend that a clear cutting-edge advantage of the fin/nanowire quantum architecture has not yet been demonstrated, i.e., still the majority of published results are coming from research with planar QW-type devices. Therefore, it is important to compare the main principles in how QDs and spin qubits are realized for the planar (2D) and fin/nanowire (1D) devices shown schematically in Figure 1c,d, with images of realized qubit architectures shown in Figure 1e,f.

The spin qubits exploit full 3D quantum confinement of the electron/hole. In planar devices using heterostructure layers, a quantum well is formed along the vertical (growth) z-direction due to the band-structure engineering at the interfaces of the neighboring layers. As shown in Figure 1c, the full 3D confinement can be established using multiple potential barriers (barrier gates) to provide confinement in the x-y direction defining QDs in a quantum well (QW). The QW is grown between two confining layers and shows properties of a 2D electron or hole gas. The barrier gates are accompanied by QDs tuning gates (plunger gates) as well as Ohmic contacts control gates to form a complex architecture of closely spaced gates. For the one-dimensional architecture reminiscent to the finFETs, the confinement in the z-direction (Figure 1d) is complemented by the potential at the sidewalls of the nanowire/fin and by potential barriers (gates) spaced along the nanowire direction. Like the planar quantum well-type devices, the potential of the barrier gates can be used to alter the shape of the QDs, while additional gates (plunger gates) can change the electrochemical potential of the QDs, and source and drain gates can control the electron/hole transport from the reservoirs to the nanowire/fin body. Generally, the voltages of all gates are controlled separately, so that the interdot electron/hole tunneling coupling and the population of the QDs can be precisely tuned for a specific spin–spin interaction.

An obvious question is about the main advantages gained from using a fin/nanowire device architecture for realizing qubits and not a planar architecture. The shift from planar to fin/nanowire FET architecture was driven by the need for enhanced electrostatic control provided by the π/Ω- or GAA gates topology [20]. This argument is still valid when better localization and control of the population of the QDs with carriers is needed. Another argument in favor of the fin/nanowire architecture would be in the number of gates used to define a QD. As seen from the schematic in Figure 1c,d, and the corresponding images in Figure 1e,f, the number of gates can be significantly reduced in a fin/nanowire device, since confinement in nanowires occurs effortlessly and is provided by their reduced dimensionality. Radial confinement, transverse to the flow of carriers, transpires due to the physical channel diameter being on the order of the characteristic length scales such as the mean free path of the carriers. This argument is supported by the demonstration of a single gate-operated QD with a Si channel cross-section of a few nm [21]. Moreover, a material such as Ge with an exciton Bohr radius approximately five times larger than that of Si can further leverage strong quantum confinement effects. Adversely, the ultimately scaled nanowire channel of the device requires a gate dielectric interface localized at only a few nm to the QDs, inflicting challenges related to trapped charges or other defects at that interface that can become source of noise and errors in the qubit’s operation.

Looking further at the choice of materials used to realize qubits, and when compared to other platforms such as superconducting qubits, semiconductor devices offer a wealth of material properties that can offer several significant advantages. As mentioned above, the electron/hole spins can be hosted in semiconductors and manipulated in a gate-defined QDs’ array that can be manufactured using the VLSI technology, underpinning today’s semiconductor industry. Hence, the advantage of the semiconductor spin qubits is in leveraging decades of technological development in the microelectronics industry, with its relentless trend toward miniaturization [8].

The unique properties of Si, most notably its oxide, resulted in Si dominating the semiconductor manufacturing with no other semiconductor in contention until major limitations such as short-channel effects or mobility degradation were seen. Some of the advancements in the device’s architecture such as the planar to finFETs transition, mentioned above, were made to continue that trend. At the time, Si was proposed as a new platform to host qubits, alternative to high electron mobility III–V 2DEGs systems such as GaAs/AlGaAs due to the possibility to use isotopically purified Si host crystal with vanishing concentrations of non-zero nuclear spins [22]. The use of a nuclear spin-free host is advantageous for the qubits’ operation because of reduced hyperfine interactions that can seriously deteriorate the spin coherence. In recent years, this property of Si, and even more in Ge, was complemented by the proposition of using holes instead of electrons due to the larger spin–orbit coupling seen for holes [23]. This will enable fast qubit manipulation, reducing the need for high magnetic fields, and possibly counteract the disadvantages of long coherence times. The absence of large magnetic structures will reduce the complexity of the devices and potentially would allow for a larger number of qubits. Moreover, it opens the possibility for all-electric spin control via electric-dipole spin resonance, where spin rotations are manipulated by oscillating electric fields. The downside of all-electrical spin control is that the required spin–orbit coupling exposes the qubit to charge noise, leading to a reduced hole spin coherence. However, there are experiments with a single hole spin confined in Si nanowire devices that pinpoint the existence of operation sweet spots where the longitudinal spin-electric susceptibility is minimized, resulting in a large enhancement of the spin coherence time [24].

**Figure 2:**
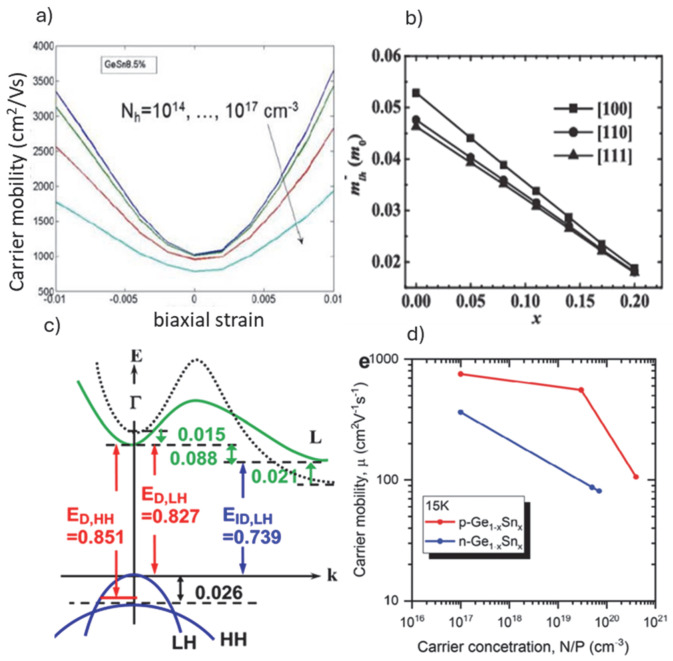
(**a**) Calculated hole mobility for GeSn (5at.% Sn), at different levels of biaxial strain and carrier concentration [27]; (**b**) light-hole effective mass calculations at increased Sn content for GeSn alloy [28]; (**c**) experimentally determined band diagrams from photoluminescence (PL) measurements at 10K along with the same theoretically predicted band diagrams (black dotted line) for 0.28% tensile-strained undoped GeSn (0.03at.% Sn) [29] and (**d**) experimentally measured carrier mobility at 15K for different carrier concentrations for 1.5% strained GeSn (10at.% Sn) substrate [31].

In addition to the larger spin–orbit coupling, the holes in group IV semiconductors allow for simpler band structure, specifically valley degeneracy for electrons, which complicates qubit definition; this is absent for holes, and excited states can be well separated in energy. This has a direct effect on the fabrication requirements, whereby better QDs definition can be achieved at relaxed dimensions for devices based on holes than with those using electrons. While Si spin hole devices have been demonstrated, Ge has the highest hole mobility, one of the properties that has put Ge onto its own quantum information route as described in a recent review [25]. Briefly, Ge can be produced with low contribution of non-zero nuclear spin isotopes but would also have the advantage of high hole mobility, given by the lower effective hole mass [26]. In fact, the Ge hole mobility and band structure can be finely modulated by proper strain engineering as well as alloying with tin (Sn) (Figure 2a,b), as shown by calculations [27,28]. Experimentally, the GeSn band structure under tensile strain was determined by low temperature photoluminescence PL measurements (Figure 2c). Additionally, devices on heterostructure-strained Ge [17] and GeSn [29] QW layers (Figure 2d) showed the existence of 2DHG and high mobility determined by Hall measurements, while FETs with gate-all-around (GAA) vertically stacked GeSn (10% Sn content) channels showed record high ON current and I_on_/I_off_ ratio [30]. These key advancements were possible due to the fast development of Ge-based fabrications that adapted many of the significant findings during the search for beyond-Si CMOS technologies as described in the next section.

## 3. Fabrications of Group IV Fin/Nanowire Quantum Devices

**Figure 3:**
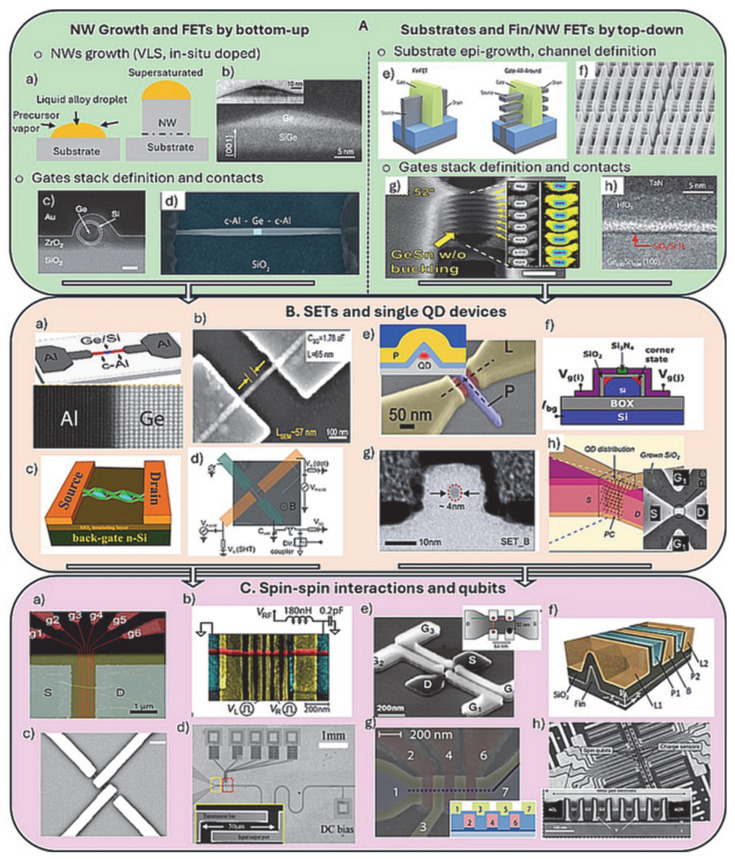
Bottom-up and top-down fabricated devices, major developments from conventional FETs to qubits. (**A**) (green box)—(**a**) Schematics of the VLS-based growth of NWs; (**b**) cross-sectional TEM image of Ge hut wires [38]; (**c**) cross-section of core–shell Si-Ge NW, showing conformal deposition of gate materials [37]; (**d**) SEM image of back-gated Al-Ge-Al device [39]; (**e**) schematics demonstrating the transition from single channel π-gate finFETs to vertically stacked nanowires GAA FET architecture [50]; (**f**) SEM image from the TSMC Si finFET technology showing massive complexity at small length scale [51]; (**g**) tilt-view SEM image of vertically stacked GeSn (10% Sn) nanosheets with GAA topology from the highest ON current p-type FET reported, the corresponding cross-sectional TEM view depicting gate materials analyzed by EDS [30]; (**h**) high-resolution TEM image of the GeSn surface passivated with Si monolayer to form better-quality GeSn-gate oxide interface and MOSFETs [52]. (**B**) (beige box)—(**a**) Schematics of core–shell Si-Ge NW in contact to Al device used to demonstrate fully transparent contacts and NW ballistic transport, with the corresponding atomic resolution Al/Ge TEM image at NW interface [46]; (**b**) SEM image of NiSi-Si nanowire devices depicting ultra-small Si QDs formed by Ni silicidation [53]; (**c**) schematics of nano-chain Ge islands device used to demonstrate SET performance in physically defined Ge islands [54]; (**d**) schematics of Ge hut device for single-shot readout of hole spins in Ge [55]; (**e**) schematics of a triangular cross-section Si fin and the corresponding SEM image of the device exemplifying QD control with a single gate [56]; (**f**) schematics of a Si nanowire on SOI depicting corner state QDs defined by split gates (Vg_i_ and Vg_j_) [57]; (**g**) cross-sectional TEM image of 3–4 nm Si-SET device operated with a single gate at room temperature [21]; (**h**) physically defined Si QD with side gates used to reveal tunable room temperature operation [58]. (**C**) (pink box)—(**a**) core–shell Si-Ge NW with multiple metallic gates by lift-off used to show gate operated QDs in an array [59]; (**b**) equivalent circuit and SEM image of a core–shell Si-Ge NW to demonstrate hole-spin qubit with high coherence times Ge [60]; (**c**) SEM image of the device used to show double QD Ge hole-spin qubit in Ge hut wires [61]; (**d**) SEM image of the whole circuitry used to demonstrate core–shell Si-Ge NW qubit to microwave photons for optical control of the qubits operation [62]; (**e**) SEM image of split-gate 2 × 2 QDs Si nanowire device used to define quadrupole array of corner QDs [63]; (**f**) schematics of a Si finFET double QD hole-spin qubit with QDs at the Si-fin apex, used to demonstrate an anisotropic exchange and above 4K operation [64]; (**g**) false-color SEM image and corresponding schematics of set of plunger (red) and barrier gates (yellow) for ambipolar QDs along the Si nanowire channel (dotted line) [65] and (**h**) top-down SEM image depicting extreme complexity of the gate terminals and corresponding TEM x-cross-section in the Si finFET region from foundry-based fabrication of qubit devices [19].

The bottom-up and top-down paradigms in the fabrication of devices based on one-dimensional semiconductors have already been reviewed [9]. Addressing the need for high performance FETs to meet the CMOS technology performance requirements, devices with grown group IV nanowires and a single gate were researched initially (Figure 3A, green box). Consequently, the same generic architecture was exploited to demonstrate device performance based on important quantum properties (Figure 3B, beige box) such as Coulomb blockade in SETs or ballistic transport with fully transparent contacts (see next section for more detail). These types of devices seeded the research that extended towards the formation of double QDs followed by specific spin–spin interactions and demonstrations of qubit operations towards quantum computation (Figure 3C, pink box). Broadly, the devices based on the top-down fabrications share common generic processing steps with the devices using grown nanowires, i.e., (i) growth and in situ doping of the QDs’ host material (fin/nanowire), (ii) conformal gate materials deposition, generally performed by ALD and (iii) contacts formation and metallization as outlined in Figure 3A–C. Below we provide more detail and use several examples to illustrate the device fabrication evolution focusing on important fabrication challenges.

Starting with the first step in the bottom-up fabrication of quantum devices (Figure 3A(left)), and specifically the nanowires growth, there are several reviews describing in detail the most used vapor–liquid–solid (VLS) mechanism, and analogs thereof, for nanowire growth [32,33]. To this end, there are two main limitations for furthering this process sequence into a viable option for large-scale quantum computing devices: (i) arrays of devices are difficult to fabricate, requiring methods for controlling the placement of the NWs on the substrate after epi-growth and/or after transfer to a device substrate [34], and (ii) during the metal catalyzed VLS-type of growth metal, most commonly gold, can diffuse throughout the whole nanowire. Gold or other metal atoms can be trapped in the nanowire and act as trap states, deteriorating the electronic properties of the QDs’ host [35]. Alternatively, self-seeded growth can provide metal-free nanowires of group IV nanowires [36]. The examples shown in Figure 3A are for nanowire structures and devices based on core–shell Ge-Si [37], Ge hut [38] and heterostructure Al-Ge nanowires [39] that were initially developed as high-performance FETs and later examined as devices functioning under quantum principles. For the core–shell NWs, there are several structural properties that are very useful for quantum transport studies. Specifically, for the Ge-Si NWs, besides the Ge surface passivation by the surrounding Si shell, there is also the possibility of introducing strain and controlling the mobility in the Ge core due to the Ge-Si lattice mismatch, as well as modulation doping of the Ge core by the shell [40]. Briefly, the Ge surface is notorious for its non-stoichiometric oxide, due to problems with non-passivated dangling bonds and trapped charges at the interface withgate oxides such as AlOx, ZrOx or else, whenttempted as substitutes of the non-stoichiometric GeOx [41]. Hence, forming Si shell in situ during growth can be highly advantageous. Moreover, Ge core of <10 nm is advantageous for controllably accommodating large strain levels without inducing extended defects such as dislocations at the Ge-Si interface [42].

The next example in Figure 3A is of the so-called Ge hut wires, grown after forming a Ge wetting layer on the Si or SiGe surface, with a resultant triangular (hut-type) cross-section. In contrast to VLS-grown structures, the hut wires are metal catalyst free; precise location and length can be controlled by using pre-patterned trenches in the Si substrate [43]. Notably, their height can be controlled to a few nm, and the added advantage of high levels of strain induced during growth make these structures suitable for studying QDs and hole states in Ge [44]. The Ge hut wires are rather unique structures; hence fabricating devices can be considered at the cross-over of bottom-up and top-down fabrications. The one-dimensional topology of the channel is defined during epitaxial growth of in-plane Ge hut wires and not by etching, while the rest of the device fabrications follow typical top-down sequence. The growth process involves epi-growth of Ge wetting layer, which after high temperature annealing transforms into one-dimensional structures, 2 nm high, exhibiting a triangular cross-sectional shape, “a hut” with a base of about 15 nm [45]. Their composition is a Ge-rich Ge/Si alloy with accumulated strain that can be controlled by further capping with Si. Using guided growth, the structures can be localized at pre-defined locations on the Si wafer, facilitating further integration in devices using fabrication sequences typical for the top-down methods.

The last example with grown nanowire devices is for Al-Ge-Al nanowire heterostructures shown at the SEM image in Figure 3A(d) from a back gated FET device [39]. The key structural component is the sharp atom scale interface between the single crystalline Al and Ge segments of the nanowire (Figure 3B(a)) [46]. The heterostructures were fabricated by a reactive thermal re-growth and exchange reaction between the single-crystalline Ge or Ge/Si core/shell nanowires and lithographically defined Al contact pads defined by metal evaporation and lift-off. The process results in self-aligned quasi one-dimensional crystalline Al leads with unique quantum device properties such as abrupt Schottky tunnel barriers, and subsequent demonstrations of single hole transport and Josephson junction transistor [39]. Moreover, the first experimental observation of room temperature quantum ballistic transport in Ge has been demonstrated with very short Ge segment Al-Ge-Al heterostructure nanowires [47]. Al-Ge contacts show also transparencies greater than 96%, allowing for the realization of a Josephson field-effect transistor and studies of the sub-gap structure that can lead the development of gatemon- and transmon-type devices [48]. In this respect another notable example is the study of high-critical magnetic field superconducting contacts between the Ge-Si core–shell NWs and NbTiN [49].

For the top-down fabrication sequence, a suitable substrate is chosen, having a device layer that will host the QDs and potentially qubits (Figure 3A(right)). All the fabrication routes follow steps that are equivalent to the π- or gate-all-around FET manufacturing routines developed at most advanced research pilot lines such as IMEC, LETI and IBM [50,51]. During these fabrications, the lithography capabilities govern the channel and gates definitions with targeted dimensions that are <30 nm, ideally in the 10 nm range. In this regard, the extreme ultraviolet lithography (EUV) has been projected to reach ultimate resolution and fidelity for sub-10 nm CMOS technology modes [52], however electron beam lithography (EBL) is currently used widely for device prototyping of such devices [66]. Most of the demonstrated devices use metal gates including magnetic materials such as Cobalt, formed by lift-off process and defined in a positive polymethylmethacrylate (PMMA)-based EBL resist [67]. While the PMMA EBL and lift-off processing modes are relatively well established, there are several clear limitations related to the density/smallest dimensions of the metal gates. Recently, an alternative negative resist (hydrogen silsesquioxane, HSQ) process has been developed using TiN as gate metal, demonstrating higher patterning fidelity alongside possibility for self-alignment of the gates to the channel [68,69]. We have further demonstrated that moving from Si substrates to epi-grown Ge heterostructure layers on Si comes with its own challenges for the EBL, limiting resolution and fidelity of the line structures obtained [66]. This is important as fin or nanowire Ge or GeSn FETs with π- or GAA gate topologies have been fabricated, and their performance showed great promise in outperforming analogous Si-based FET architectures. However, such devices have not yet made the transition to devices showing spin qubits operation. Nevertheless, we postulate that the path to fin/nanowire Ge or more challenging GeSn qubit devices fabricated by fully CMOS compatible routines can be very short. This is due to the fast maturing of the Ge and GeSn devices fabrication technology [70]. Initially, the Ge and GeSn devices were researched with grown nanowires and associated surface passivation/treatment methods [71], ALD of high-k oxides [38] and the reactive re-growth of contact materials such as germanides and stanogermanides [72]. In parallel, the ongoing research in planar Ge and GeSn MOSFETs and photonic devices has been strongly advanced by the ever-improving growth capabilities for strained Ge and GeSn heterostructure substrates [25].

From a fabrication perspective, the complete realization of the potential and fault-tolerant qubit operation requires full optimization of the whole process flow. This is particularly valid for the top-down fabrication sequences, where steps such as gate materials and contacts formation, depositions and etching steps can introduce different sources of trapped charges, defects and inhomogeneities at the interfaces that can be detrimental to the final fault-tolerant qubit operation. This is supported by a recent investigation of process variability and qubits fidelity validation for devices fabricated in a foundry [19]. While all solid-state systems are subjected to some materials noise and disorder, spin qubits probe these imperfections at nearly the atomic scale, imposing sturdier fabrication requirements than those for conventional logic devices at their ever-decreasing size. The ongoing development of the group IV spin-based quantum devices roadmap can certainly adopt many of these developments when proposing solutions for fault tolerance and large number of qubits operations. In the next section we provide examples with group IV nanowire/fin devices that underpinned the development and established baseline operations with QDs, furthering the ongoing realization of group IV materials for hosting qubits.

## 4. Mesoscopic Physics with Group IV Nanowire/Fin Devices, Quantum Dot Devices and Spin–Spin Interactions

**Figure 4:**
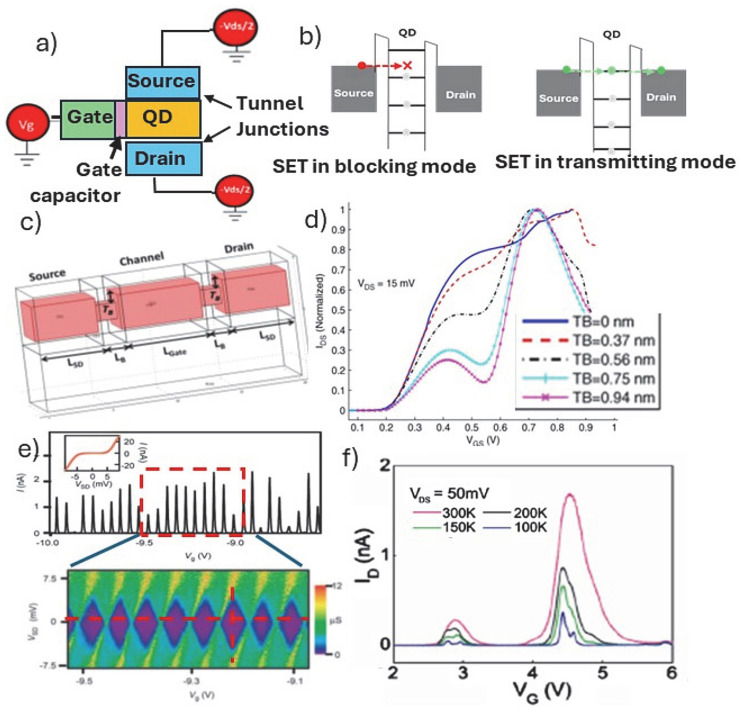
(**a**) Schematics of a SET device featuring a Coulomb Island with a QD, associated tunnel junctions to the source and drain and a gate with gate capacitor at the gate oxide–QD interface; (**b**) schematics of the energy levels for a blocking and transmitting SET; (**c**) schematic of the π-gate Si nanowire FET on SOI (in pink) depicting tunnel barrier constructions (TB) and channel with 3 nm × 3 nm cross-section and 10 nm length that was used to calculate the I_SD_-V_G_ plot shown in (**d**) for different TB sizes [73]; (**e**) Coulomb blockade oscillations seen in the I_SD_/V_G_ plot measured for a core–shell Ge/Si nanowire (inset: I_SD_/V_SD_ plot, at V_G_ = 9.35 V), both plots are taken across the marked dotted lines in corresponding stability diagram (I_SD_-V_G_) depicting uniform Columb diamonds [42]; (**f**) temperature dependent I_SD_-V_G_, plots measured for a π-gate Si nanowire FET on SOI fabricated with 3–4 nm sized channel [21].

While the operation of the spin qubits and the extraction of the figures of merit describing the fidelity of spin–spin interactions in terms of coherence times, charge noise, range of interactions, fault tolerant operation, controlled noise, etc. can be fully described by the quantum effects and theory, there are important principal considerations that can be used to set up the foundations. One of the underpinning effects and a type of device is the Coulomb blockade and the single electron transistor (SET) devices. The operation of the SET is based on the principle of the Coulomb blockade effect which arises from the discrete nature of charge and the electrostatic repulsion between electrons or holes. It consists of an island (defined in the channel of a conventional FET) that is coupled to source and drain charge reservoirs via tunnel barriers; therefore, sometimes such devices are referred to as tunnel SETs (Figure 4a). There are different ways how the island and the barriers are realized. One can use insulating tunnel barriers as shown in Figure 3B(c) for a chain of Ge islands [54], or at the tips of two doped Si on insulator source/drain regions that after oxidation form a Si island as seen in Figure 3B(h), or by using electrically induced potential barriers along a fin or nanowire [19]. Nonetheless, if the island’s electrons/holes are confined in three dimensions, a quantum dot is formed such that discrete energy levels are obtained (Figure 4b). The energy levels within the QDs typically exhibit evenly spaced intervals of ΔE giving rise to a self-capacitance C of the island, defined as C = e^2^/ΔE (where e is electron charge and ΔE is the spacing between the energy levels in the QD). Generally, realizing Coulomb blockade, for V_SD_ < e/C, is governed by the ability of confining barriers to trap electrons/holes in a QD and the electrostatica repulsion of any other electron/hole attempting to join that dot. Considering the total capacitance of the QD, one can establish the charging energy, the energy required to add one electron/hole to an electrostatically protected island, E_c_ = e^2^/C_t_, which should be well above the thermal energy (k_B_T) in both the source contact and the island. Commonly, the changes in the magnitude of the gate voltages seen during the formation of the Coulomb diamonds are used to calculate the QD capacitance and the charging energy, where the total QD capacitance considers the capacitance at the gate and the tunnel barriers. In all cases, uniformly shaped diamonds that are constant over several charge transitions are targeted (Figure 4e), validating the SET constant interaction model.

In an attempt to follow the transition from the operation of single gate nanowire FET to a confined QD island, an electrical potential model has been developed for a device with a generic architecture based on a Si-nanowire (3 nm in width and 10 nm long), and two barrier constrictions are applied that allow for a QD formation along the wire as shown in Figure 4c [73]. Using the 3D potential models one can demonstrate that the transport regime can transition from 1-D to 0-D transistor performance, and it is directly related to the width of the constrictions and the source/drain (V_SD_) bias. At low V_SD_ and low temperature, the transport characteristics seen on the I_SD_-V_GS_ start to exhibit oscillations where the positions of oscillation peaks are associated with energy levels in the quantum dot structure due to 3-D confinement (Figure 4d). There is a clear dependence on the size of the barrier constrictions responsible for the 0-D island formation, and the appearance of oscillations in I_SD_ is a function of applied gate potential (V_GS_) as demonstrated in Figure 4d. Supportive to this simulated device performance is the experimental I_SD_-V_GS_ data obtained using Si-FET device with a single gate. Figure 4f depicts I_SD_ oscillations at low temperatures that were made possible by a delicate dry etching and Si oxidation process to reduce the size of the channel forming a SET island of only 3–4 nm [73]. The corresponding TEM cross-section is shown in Figure 3B(g). The claim was that this key step enabled the formation of identical tunnel barriers in a self-aligned manner. Additionally, the π-gate wraps most of the way around the active channel, thus ensuring better capacitive control over the function of Coulomb island.

In parallel, grown nanowires such as core–shell Ge-Si nanowires have shown much promise, as seen in several early works where high-performance FETs for CMOS logic were targeted; an example of such a device is depicted in Figure 3A(c) [37]. The advantage with the core–shell morphology is in the specifically engineered band structure that allows for realization of oscillations in the I_SD_ as shown in Figure 4e [42]. Notably, the valence band offset between Ge and Si at the heterostructure interface of 500 meV serves as a confinement potential for the quantum well, and free holes will accumulate in the Ge channel when the Fermi level lies below the valance band edge of the Ge core. Moreover, the thin Si shell provides a suitable interface for reducing holes scattering in the Ge channel and for reducing the complications with the Ge oxide surface when forming the gate oxide. Using these key features of the grown core–shell Ge-Si nanowires and just one π-gate, a well-defined Coulomb blockade stability diagram (Figure 4e) and relatively high extracted charging energy have been reported.

**Figure 5:**
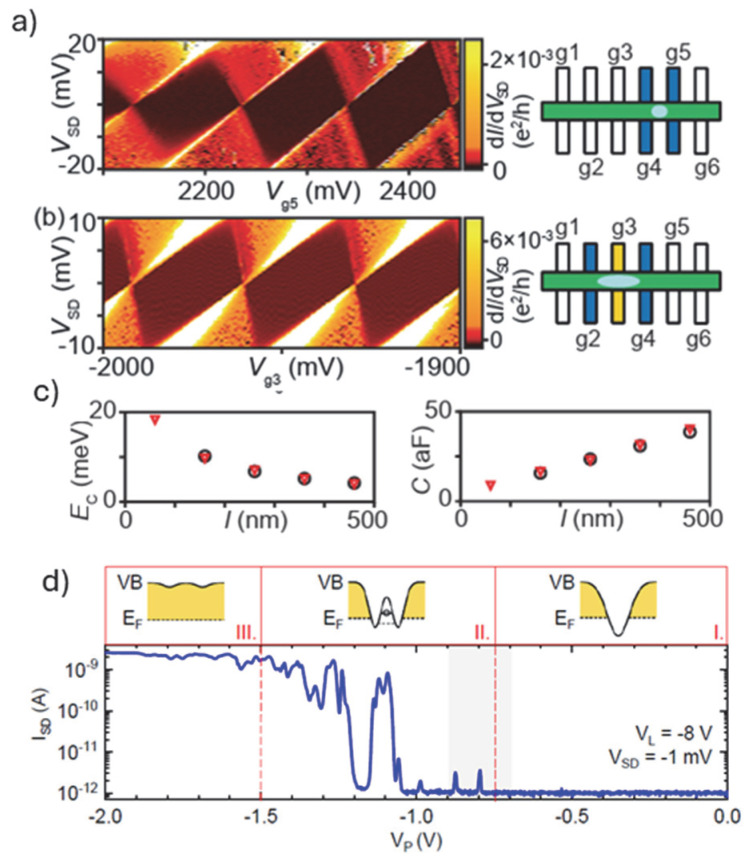
Charge stability diagrams and corresponding schematics of gate-defined single QDs formed in a core–shell Ge/Si grown nanowire (the corresponding SEM image of the device is shown in Figure 3C(a)), with (**a**) zero and (**b**) one plunger gate between the barrier gates indicated in blue, whereas light blue shows the approximate size of the formed QD [59]; (**c**) corresponding charging energy E_C_ (**left**) and total capacitance C_t_ (**right**) of the dot plotted versus the dot length l (nm) and (**d**) source/drain current I_SD_ versus plunger gate voltage VP in the low-bias regime (V_SD_ = −1 mV) for a ambipolar device with Schottky NiSi contacts formed using a triangular cross-sectional Si channel (SEM image in Figure 3B(e)). Top panel: Sketches of the real space band alignment in the vicinity of the plunger gate for the observed three different conductance regimes [56].

For gate-defined QDs and their use as qubits, multiple sets of gates (barrier and plunger gates) are required as depicted in the SEM image in Figure 3C(a). The gates were defined by lift-off on top of a core–shell Ge-Si nanowire channel with its engineered quantum properties as described above. Initially, one can use this architecture to rationalize how well protected (electrostatically) the quantum dots are [59]. Figure 5a shows a charge stability diagram obtained by using only two barrier gates (see schematics in Figure 5a(right)). The shape of the Coulomb diamonds measured are non-uniform, while Figure 5b demonstrates operation under the constant interaction model when an additional plunger gate is included. Besides the uniform shape of all Coulomb diamonds measured, the Coulomb peaks spacing along V_SD_ = 0 mV indicates a constant gate capacitance over several charge transitions. This “ideal” behavior is attributed to the use of a dedicated plunger gate alongside the barrier gates as depicted in the corresponding schematics. Moreover, one can estimate separately the capacitance at the gate (plunger) and at the tunnel junctions to the QD controlled by the barrier gates. Provided that plunger gate capacitances are constant, the total QD capacitance scales linearly with the distance between the barrier gates, providing an indication about the size of the QD as plotted in Figure 5c. Correspondingly, the charging energy is scaling down with increasing the distance between the barrier gates (Figure 5c(left)). Table 1 provides a summary of extracted total capacitance and charge energy values from charge stability diagrams and corresponding references comparing devices with grown nanowires and top-down fabricated devices. Possible room temperature operation is highlighted.

Another way to realize Coulomb blockade and SET performance is to establish tunnel barriers by forming Schottky junctions between the source/drain and the Coulomb island. By tuning the silicidation process within a Si nanowire, ultrasmall Si QDs have been realized as seen in Figure 3B(b) for about 40 nm QD [53]. Using the same approach there were claims for QDs down to about 6 nm, having charging energy of 120 meV, well above the energy required for room temperature operation. Similarly, using Si fins and reactive growth of Ni-silicide source/drain regions, an ambipolar SET device with just one π-gate has been realized (Figure 3B(e)) [56]. By choosing a mid-gap silicide, ambipolar operation is realized in a simple, highly compact design, as no complementary charge reservoirs are required. The band alignment around the plunger gate depicting three distinct transport regimes and the corresponding I_SD_ versus plunger gate voltage is shown in Figure 5d. Later work has extended the approach of forming metal-semiconductor source/drain regions with single crystalline interfaces. As mentioned in the previous section, notable example includes the formation of atomically abrupt Al nanowire segments to core–shell nanowire Ge/Si interfaces (Figure 3B(a)). The intricate band structure of the core–shell Ge-Si grown nanowires and the atomically controlled interface allowed the realization of one-dimensional electron/hole gas (1DEG) with ballistic transport and no significant scattering centers inside the channel up to a channel length of at least 500 nm [47].

The importance of the work centered around the core–shell Ge-Si gown nanowires is further exemplified in Figure 3C(b,d) [60,62]. This work builds upon a decade of research on the Ge/Si core–shell wires—following the pathway from high-performance FETs to devices showing 1DHG and ballistic [42] transport to hole-spin qubits with high coherence times [60] and further coupling to a microwave transmission line resonator for a controllable and coherent light–matter interface [62]. Specifically, in the latter example, local barrier gates along the nanowire are defined (Figure 3C(d)), and a two-level system is defined close to the charge transition degeneracy between the adjacent QDs due to the existence of tunnel coupling. The charge qubit energy can be tuned relative to the cavity photon level using the gates, thus switching on and off the coupling.

Similarly, the pathway of the Ge hut wires to quantum devices has seen prolific development. Capitalizing on the strong spin–orbit coupling predicted for holes, the Ge hut wire devices were used for the first time to demonstrate single-shot readout experiments with holes; a circuit diagram and image of the single-gate device are shown in Figure 3B(d) [55]. This was extended into double QDs architecture (Figure 3C(c)), where the Pauli spin blockade and the electric dipole spin resonance (EDSR) technique were used to demonstrate the manipulation of single holes, localized into the QDs [61]. Most importantly, a dephasing time (T*_2_) of 130 ns was measured that exceeded the values measured for electrons in Si.

**Figure 6:**
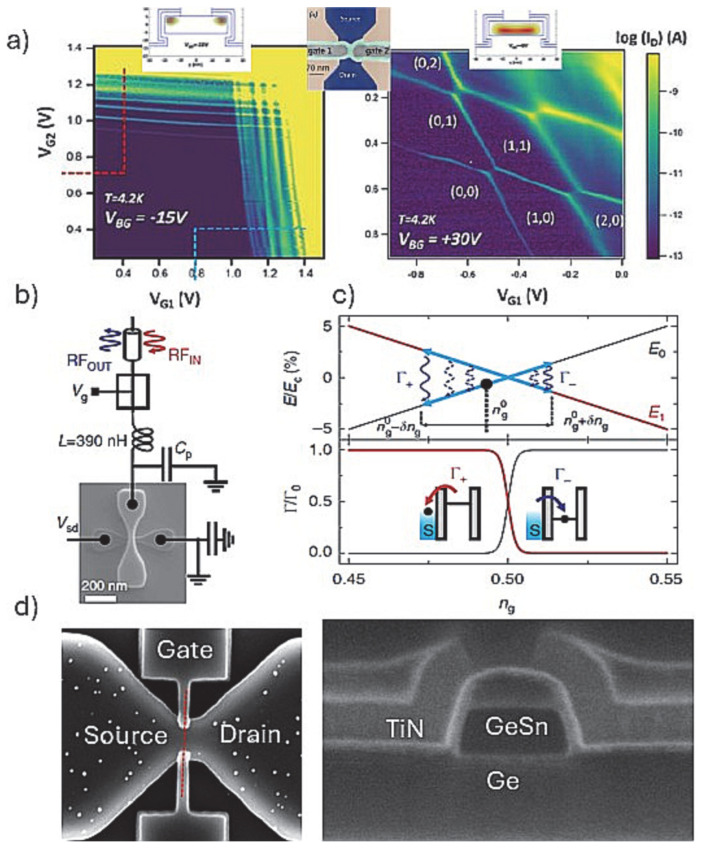
(**a**) Charge stability diagrams for a double QD defined by using two split gates (face-to-face) in a Si nanowire fabricated on SOI; the diagrams are taken with two different back gate (V_BG_) voltages (**left** and **right**), demonstrating tunability of the Coulomb interactions [57]. Depicted in the insets are simulated potential distribution maps at the two different V_BG_ accordance with interactions observed; shown also is a color-coded SEM image of the device topology outlining the split gates configuration; (**b**) SEM image and the associated RF circuit for a similar device (note the use of only one gate and larger width of the Si fin) used to define corner state QDs in a single gated finFET [74]; (**c**) corresponding energy band diagram as a function of gate voltage n_g_ (**top panel**) for the device in (**c**). The initial detuning position is set by n^0^_g_ and δn_g_ is the amplitude of the RF excitation. Γ+/− represents the tunneling into (out of) the dot; calculated 3D to 0D tunnel rates as a function of n_g_ (**bottom panel**) for charging energy E_C_ = 15 meV and T = 100 mK; (**d**) SEM images of a split gate device fabricated in GeSn/Ge heterostructure substrate, featuring TiN gate and AlO_x_ gate dielectric as shown on the cross-sectional image on the right [69].

The top-down FETs fabrication pathway (Figure 3(right hand side)) has mostly developed around using the Si channel for hosting QDs and expanded in seminal demonstrations gate-defined fin/nanowire qubit devices as discussed below. While determining the localization and size of the QDs in the FET channel is still a matter of debate, there are reports where the QDs were localized at the apex of a triangular [56] or the corners of rectangular shaped Si channel [74] by a single gate as seen in Figure 3B(e,f). The device performance was related to the operation of a π-gate FET where channel formation occurs at the edges of the channel. In the case of the triangularly shaped nanowire device, the total capacitance of the QD implies a much larger QD size than the physical size of the Si fin apex. This as well as the inferior characteristics seen in the charge stability diagram suggest that the device performance can be affected by charge-trapping defects. It also shows the inherited ambiguity in the QDs formation induced potential landscape near the channel, forming disorder. The numerical simulations for a rectangular Si channel show that due to the side wall roughness introduced during fabrication, a delocalization of the conduction channels at the edges can occur, forming non-intentional corner sate QDs even with a single π-gate. Arguably, this effect is related to the width of the rectangular channel, i.e., in the narrow-channel device the carriers are located under the gates close to the top face of the Si nanowire. For larger channel widths, however, two pronounced potential minima develop at the upper nanowire corners, leading to a pair of clearly distinct QDs [57]. Follow-up works showed that the corner state QDs formation can be better controlled by the set of gates covering just the edges of the channel (Figure 6a). This is beneficial as independent electrostatic control of the two corner dots can be achieved. The inter-dot coupling, mediated by tunneling and Coulomb interaction, can be further tuned by means of a global gate (back gate on SOI). The splitting of the single gate into two face-to-face gates, however, requires delicate fabrication going beyond π-gates formation as demonstrated by using the self-aligned gates patterning process [68]. We have demonstrated a similar process sequence for GeSn structures using a combination of selective Ge to GeSn etching and ALD of gate stack depositions (Figure 6d) [69]. Most importantly, the tunability of the corner state QDs can be demonstrated by controlling the inter-dot capacitive. This is seen in the charge stability diagrams at different back gate voltages and by the numerical simulations of the single-electron states in the channel cross-section (Figure 6a and insets).

The specific interaction of the double QD formed at the corners of the Si nanowire was systematically studied using a single-shot readout (Figure 6b) [74]. Using a relatively simple architecture with just one π-gate coupled to a RF reflectometry circuit, the sensitivity limits of resonant readout has been benchmarked. This gate-based sensing method based on the spin-to-charge conversion is performed by using one of the corner dots as a sensor dot that is connected to the RF circuit. The gate sensor alleviates the need for an external electrometer and simplifies the qubit architecture. As mentioned above, in such corner state, double QDs form under a single gate due to the potential irregularities along the transport direction. The operation is depicted in Figure 6c and further explained in the figure caption. Briefly, as the gate voltage is changed via the RF excitations, the tunning of the QDs is controlled by having carriers tunnel in and out of the QD. Hence, the tunneling rates of carriers from a reservoir (3D) to the QD (OD) can be calculated as a function of gate voltage changes initiated by the RF excitations. Similarly, the operation of a 2 × 2 QDs array (Figure 3C(e)) was studied by applying gate-voltage excitations on one of the gate electrodes coupled to a RF circuit, demonstrating exchange of electrons by spatial permutation [63]. The array is reconfigurable in situ to realize various multi-dot configurations with an additional overall adjustability of tunneling times by a global top gate electrode [75]. For all Si platforms discussed above, exploiting either fin or NW FET channels with electron or holes spin states defined, fast single-shot readout of spins can be applied for accurate qubit measurements. At few-kelvin temperatures, this can be realized using a DQD charge sensor that exploits tunneling between two quantized states. This technique is more resilient against temperature than a single-sensor QD and provides a high-fidelity single-shot readout resolution of <1 μs, which is fast compared with spin lifetime. These examples advocate that the resonant gate-based readout has advantages over external electrometers both in terms of reduction of circuit elements as well as absolute charge sensitivity [74].

**Figure 7:**
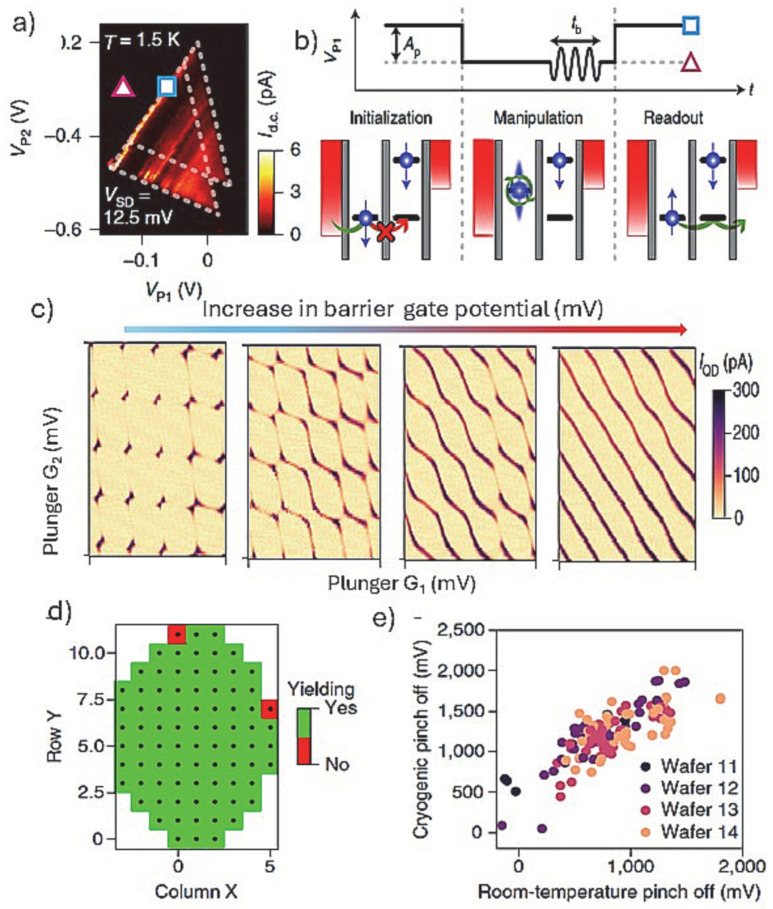
(**a**) Measurement of a spin-blocked pair of bias triangles realized by using a device based on a Si-fin hosting a double QD qubit as shown in Figure 3C(g) [65]. The blue square and pink triangle mark the qubit initialization/readout and manipulation point, respectively; (**b**) the corresponding schematic of the spin manipulation cycle with the pulse scheme; (**c**) charge stability diagrams for a double QD formed under plunger gates, showing good control over the interdot tunnel coupling as the barrier gate potential is increased; the electron microscopy images of a representative device are shown in Figure 3C(h) of multi-gate Si-fin qubit devices fabricated at a microfabrication foundry [19]. (**d**) corresponding room-temperature yield map for all devices on a wafer and (**e**) correlation map for threshold voltages at room temperature versus low temperatures for all the gates from all the samples from the wafers that have been cooled.

While the devices discussed in Figure 6 use corner state QDs, the most common type of spin–spin interactions studied are along the fin/nanowire length, similarly to the devices using grown nanowires. For example, a set of gates were used to define QDs along a rectangular Si nanowire channel, and by using an RF reflectometry circuit, a real-time detection of spin-selective tunneling to a reservoir QD was demonstrated [24]. The results reveal the existence of operational sweet spots where the impact of charge noise is minimized while preserving an efficient electric-dipole spin control. There are other reports that use higher numbers of gates, i.e., barrier and plunger gates as depicted in Figure 3C(f), positioned along a triangularly shaped Si finFET channel [76]. Figure 7a shows the measurement of a spin-blocked pair of bias triangles, formed by negatively biasing the gates and forming an accumulation-mode hole double QD, hosting two individual spins. Nominally, the ±1/2 spin is assigned to the two lowest-energy hole states, which for one-dimensional-like hole systems can have large contributions of both heavy-hole and light-hole basis states [77]. The measurements consist of three stages (Figure 7b): First, the two-hole spins are initialized in a polarized spin state through Pauli spin blockade. Then, the system is pulsed into Coulomb blockade, where the microwave signal is applied. Finally, in the readout stage, a current is detected if the spins are antiparallel, such that one hole can tunnel into the neighboring QD and exit into the nearby reservoir. Using this architecture and measurement, an anisotropic exchange interaction of two hole-spin qubits was demonstrated with a conditional spin-flip in 24 ns. The magnetic resonance exchange demonstrated was anisotropic because of the stronger spin–orbit interaction for holes. Upon tunneling from one quantum dot to the other, the spin is rotated by almost 180 degrees [64]. With this approach, robust spin–spin interaction was demonstrated with a potential for a large-scale qubits interaction. Moreover, due to the stronger spin–orbit interaction in the Si finFET hole spin device, it has demonstrated high-temperature operation (about 4K) with fast spin rotations and weak hyperfine couplings.

The Si qubits operation with devices made by foundry-based fabrications has provided a flexible route to demonstrate single to multiple QDs arrays with key performance indicators. A notable example includes ambipolar conventional FETs and ambipolar gate-defined quantum dots achieved by extending a gate over an intrinsic silicon channel using highly doped n-type and p-type terminals formed by ion implantation (Figure 3C(g)) [65]. The ambipolar carrier reservoirs to the Si channel allows, with the same gates, double quantum dots with either holes or electrons to be exploited. The operation of the double QD qubit was read by dispersive gate-based reflectometry to sense the inter-dot charge transition of both electron and hole double quantum dots. In this context, the fidelity of the nanoscale gate patterns with excellent yield, achieved by fully industrial Si finFET processing in isotopically rich ^28^Si has proven to be a crucial feature for fault-tolerant two-qubit gates [19]. SEM images and cross-sectional TEM images of the array of gates are shown in Figure 3C(h). The corresponding charge stability diagrams (Figure 7c) for a double QD formed under plunger gates, showing good control over the interdot tunnel coupling as the barrier gate potential is increased. Even in the multi-electron regime, the quantum dots allow good tunnel barrier control and qubit operation. Figure 7d shows a map that indicates the device yield on a wafer with only a few devices failing near the edges of the wafer. Moreover, an almost linear relationship between the threshold voltages measured at room temperature and those measured at low temperature (5 K and below) was determined (Figure 7e). Remarkably, only 21 out of 1050 gates were not working, indicating that room-temperature measurements can be used to pre-select samples to cool down for QD analysis. This is a rare case where such high number QD device-yield analysis is presented, advocating that fully CMOS compatible fabrications (e.g., gates definition by etching and not metal lift-off) are fundamental for high-yield qubits.

## 5. Outlook and Conclusions

Finally, we provide our perspective on the role of the gate-defined qubits in semiconductors by contrasting across other platforms, as well as indicators such as coherence times, fabrication maturity, scenarios for scale-up and manufacturing a large number (>1000) of qubits and co-integration to conventional CMOS cryogenic control/readout electronics on the same platform (Table 2). Unlike superconducting circuits, ion traps and color centers, the early measured coherence for the semiconductor spin qubits has been only moderate commonly below 100 ns [78]. By using nuclear spin-free hosts, such as ^29^Si, the dephasing time (T*_2_), a type of de-coherence used to describe how long a qubit can maintain its quantum phase information before losing it due to environmental noise and fluctuations, measured values exceeded 100 μs, approaching millisecond range, as shown in Table 2 [8]. While these values can be directly linked to overcoming the known sources of noise and de-coherence, as discussed below, the superconducting qubits, being one of the most mature qubit platforms, commonly demonstrate T*_2_ in the few 100s’ μs range, with two-qubit operation fidelity often above 99.5% [79]. Similarly, the color centers, typically in the form of a nitrogen vacancy (NV) center in diamond have T*_2_ at room temperature in the tens of microseconds, with higher values demonstrated with isotopically purified ^12^C [80] and divacancy in CVD SiC [81]. These values, however, are well overpassed by qubits with trapped atomic ions. Working in a vacuum, and not embedded in noisy solid-state environment, the qubits with ion traps have much larger coherence times approaching tens of seconds for single qubits, with unbeatable fidelities of over 99.99% [82]. However, micro-electrodes used in ion trap devices, although readily obtainable by common microfabrication, have limitations with respect to miniaturization, hence the scenarios for scale-up and mass-production are restricted. A possible option for mass manufacturing can be 3D-printed micro ion trap technology [83]. Although the color center qubits themselves require no dedicated fabrication, the assembly into functional devices to allow for local control and addressing is, however, needed, and it is very challenging due to the difficulties with microfabricating diamond structures [84]. Alternatives include color centers in Si, but the maturity of this platform is still low. The superconducting transmon qubits, being at the forefront of the qubit technology for information processing, rely on capacitors with a large footprint of ∼10^5^ μm^2^. The large footprint of the devices is needed to acquire sufficient capacitance, but they suffer from surface losses that can be a major source of de-coherence [7]. In fact, the major advancements that made superconducting qubits the most serious contender for quantum supremacy are related to either improving coherence through microwave engineering to avoid losses associated with surfaces and interfaces [85] and minimizing the effects of thermal noise and quasiparticles [86]. However, little progress has been made in addressing the microscopic source of loss and noise, specifically in the context of miniaturization of the circuitry. Hence, the coherence values for conventional Al–oxide–Al transmon qubits have not improved drastically in the last 10 years. This gap has been recently reviewed, with the prospects of using new materials [87]. However, many of the proposed options, such as Nb and Ta superconductors and 2D materials [88], may suffer from limited CMOS fabrication compatibility and scalable processability. A notable example of the highest reported superconducting qubit dephasing time is approximating 1 ms, with a process that is CMOS compatible yet at the micron scale [89].

On the other hand, the many years of perfecting CMOS manufacturing and the continued push to miniaturization resulted in transistor devices with unsurpassable density approaching several billion per cm^2^ and very high complexity [90]. For more than a decade, new channel materials (Ge and its alloys, specifically), gate materials and interconnects were introduced onto the same Si platform by using CMOS compatible transistor processing and integration [91]. In parallel, high coherence and high-fidelity control of spins in Si has been demonstrated, and the Ge quantum pathway has been introduced with the main benefits outlined in Section 2. Most importantly, the best performing qubit devices were manufactured using the machinery of the same top-down CMOS technology [8], including the advancements in the fin/nanowire fabrications. While the qubit devices using 2DEG or 2DHG layers are most researched and showed high qubit fidelity and operation versatility, the devices with top-down Si fin/nanowires have shown their great promise too [21,74,75]. However, the Ge-based fin/nanowire qubit devices are not yet studied, but devices with grown Ge nanostructures, manufactured by top-down methods, are well studied and showed their importance in [42,59]. Nonetheless, many of the sources of de-coherence and noise, such as unwanted contribution from non-zero nuclei, trapped charges, defects and inhomogeneities at the interfaces, dopant and crystal abruptness of the junctions, etc., have been thoroughly examined over the years. Not all these studies have been directly related to quantum devices, but all have enlarged drastically both material understanding and device fabrication to the level where the spin-based qubits in semiconductors have the potential to reach levels as good as or better than superconducting hosts. This can be achieved via (i) use of nuclear spin-free hosts by isotope-purified growth such as demonstrated for Si and Ge (ref); (ii) use of hosts with lower disorder, reduced defects and controlled strain via ever improved Ge, SiGe and GeSn on Si heterointerface growth and (iii) development of fabrication routines that are well-established in a foundry-based VLSI production including fin and nanowire fabrication technologies. This will not only result in lowering the overhead of manufacturing but also provide a direct path for co-integration of quantum components with conventional CMOS-based cryogenic control/readout electronics. Moreover, because of the variation in the operational parameters for a given qubit’s host material (most likely isotopically purified nuclear spin-free) and type of qubit architecture, the fidelity of operation is directly related to atom-scale imperfections in the fabrications, including the growth of the starting substrates, etching or deposition methods, and corresponding crystal/chemical interfaces and surfaces (Table 2). Although some of the shortcomings from inferior fabrications can be alleviated by clever design of the circuitry and use of in-build error corrections, on-chip training, etc., the recent publications looking at statistical evolution of the qubits performance with >1000 s devices fabricated by foundry processing [19] are advocating for directing the efforts towards demonstrating that high-fidelity fabrications = high fidelity qubit operation.

**Table 2:** Qubit platforms with associated performance (dephasing time) and device processing including potential for scale-up and co-integration to CMOS platforms.

Qubit Platform	Qubit Dephasing Time (T*_2_,) ^	Type and Maturity of Fabrications *	Known Sources of Noise and Proposed Processing Solutions #	Potential for Scale-Up and Co-Integration with Conventional CMOS	Ref.
superconducting circuits	100 s μs, (0.5 ms)	micron-scale, CMOS-compatible; *high*	impurities at surfaces, dielectric loss, quasiparticles; *new materials with limited CMOS compatibility*	Limited by micron-scale	[79,85,86,87,88,89]
gate defined QDs in semiconductors	100 s ns, (84 μs)	nano-scale, fully CMOS-compatible; *high/moderate*	nuclear spin, charge trapping, defects at surfaces and interfaces; *isotope purification, atom-scale fabrications and new materials with CMOS compatibility*	High and fully commensurable to CMOS technology	[8,19,78]
colour centres	10 s μs (0.5 ms)	micron-scale, partially CMOS-compatible; *moderate*	Paramagnetic impurities defects at surfaces and interfaces; *Atom-scale fabrications and new materials*	Limited by micron-scale	[80,81,92]
ion traps	1 ms (10 s s)	micron-scale, partially CMOS-compatible; *moderate*	Not significant	Limited by micron-scale, restricted CMOS compatibility	[82,83]

^ Qubit dephasing time—commonly reported and highest reported in brackets; * maturity of fabrications—shown in italics; # proposed processing solutions—shown in italics.

Underpinned by the large potential for fast scale-up via the VLSI fabrications, the spin-based qubits with semiconductor QDs comply favorably with system-level considerations such as quantum sensing, networking and edge computing that are increasingly important for the future of scalable quantum technologies. In this regard, recent perspectives on the concept of a quantum edge simulator highlight the need for co-design approaches that integrate qubit devices with quantum sensors and edge processing nodes. Such approaches not only complement device-level advances but also underscore the interdisciplinary challenges of connecting fabrication and materials bottlenecks with system-level architectures, thus linking transistor-derived qubits to broader distributed quantum infrastructures.

In conclusion, herein we review the latest advancements in group IV one-dimensional semiconductors for qubit devices, making parallel comparisons to the development of transistor technology and advantages thereof. In Section 2, we have discussed the foundational principles of quantum computing before delving into materials, architectures and fabrication routes, separately, by comparing the bottom-up and top-down approaches (Section 3). We demonstrate that due to easily tunable composition, crystal/interface quality and relatively less demanding fabrications, the study of grown nanowires such as core–shell Ge-Si and Ge hut wires has created a very fruitful field for studying unique and foundational quantum phenomena. On the other hand, the CMOS compatible, top-down fabricated devices, mainly as Si fin/NW FET architecture, showed their potential for scaling up the number of qubits while providing ways for VLSI and co-integration with conventional CMOS. We further revisited the mesoscopic physics and the fundamental properties of the hosts for defining QDs (Section 4), where we emphasized the importance of the early studies related to QDs formation bridging to the operation of SETs and their performance indicators, such as Coulomb blockade, ballistic transport, etc., that seeded the expanding research in the field of group IV fin/NW FET qubit devices. Finally, we provided our perspective on the future challenges and opportunities by contrasting the semiconductor spin qubits to other qubit platforms.

## Figures and Tables

**Table 1:** Summary of total QD capacitance and charging energy for various types of single QD devices as described.

Total Dot Capacitance (aF)	Charging Energy Ec (meV)	Type of Device	Qubit Dephasing Time (T*_2_)	Ref.
122.7	1.3	Core–shell Si/Ge NW-single gate		[42]
43.2	3.7	Core–shell Si/Ge NW-single gate		[42]
8.8	18.3	Core–shell Si/Ge NW-barrier/plunger gates		[59]
16.0	10.2	Core–shell Si/Ge NW-barrier/plunger gates		[59]
118	1.7	Core–shell Si/Ge NW-barrier/plunger gates	180 ns	[60]
38.6	4.1	NiSi-70 nm Si-NiSi NW-back gate		[53]
1.3	123.0	NiSi-6 nm Si-NiSi NW-back gate		[53]
0.3	500.0	Chain of 5–10 nm Ge crystals-back gate		[54]
0.4	400.0	SOI, 2–3 nm Si-single gate		[21]
1.1	145.5	SOI, 4–5 nm Si-single gate		[21]
19.0	8.6	SOI, corner QD-single gate		[74]
19.0	8.6	SOI, corner QD-split gate		[75]
15.1	10.6	SOI, 40 nm Si-barrier/plunger gates		[65]
10.0	16.0	Si-fin-apex QD-single gate		[57]
20.0	8.0	Si-fin-apex QD-barrier/plunger gates		[68]
		Si-fin-barrier/plunger gates	0.2 μs	[76]
		Si-fin-barrier/plunger gates-corner dots	84 μs	[24]
		Si-fin-barrier/plunger gates	24 μs	[19]
		SiGe/Ge/SiGe-planar device	44μs	[18]

## Data Availability

No new data were created or analyzed in this study.
